# *QuickStats:* Percentage* of Residential Care Communities^†^ That Use Electronic Health Records,^§^ by Community Bed Size^¶^ — United States, 2016

**DOI:** 10.15585/mmwr.mm6704a8

**Published:** 2018-02-02

**Figure:**
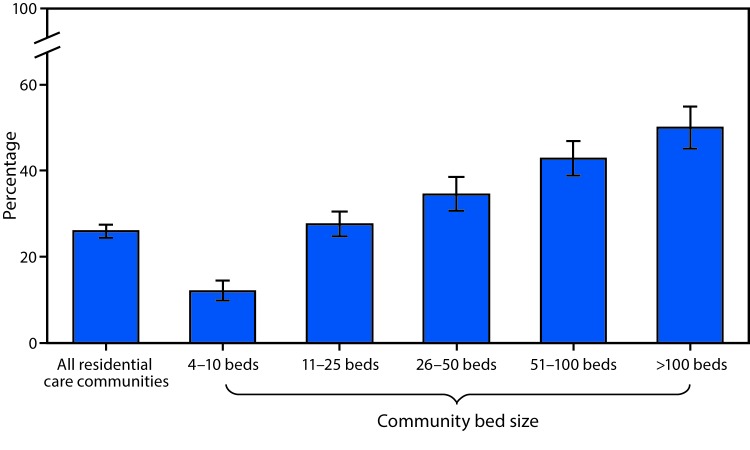
In 2016, one fourth (26%) of residential care communities used electronic health records (EHRs). The percentage of communities that used EHRs increased with community bed size. The percentage was 12% in communities with 4–10 beds, 28% with 11–25 beds, 35% with 26–50 beds, 43% with 51–100 beds, and 50% with >100 beds using EHRs.

**Source:** National Study of Long-Term Care Providers, 2016. https://www.cdc.gov/nchs/nsltcp/nsltcp_rdc.htm.

